# Agreement on the prescription of antimicrobial drugs

**DOI:** 10.1186/s12879-015-0992-y

**Published:** 2015-06-30

**Authors:** Eduardo Casaroto, Alexandre R. Marra, Thiago Zinsly Sampaio Camargo, Ana Rita Araújo de Souza, Carlos Eduardo Saldanha de Almeida, Elizia Piassi Pedroti, Elivane da Silva Victor, Oscar Fernando Pavão dos Santos, Michael B. Edmond, Alexandre Holthausen Campos

**Affiliations:** Intensive Care Unit, Hospital Israelita Albert Einstein, São Paulo, Brazil; Division of Medical Practice, Hospital Israelita Albert Einstein, Av. Albert Einstein, 627/701 – 1st floor – Bloco A1 – Room 108 Morumbi, 05651-901 São Paulo, Brazil; Instituto Israelita de Ensino e Pesquisa Albert Einstein, Hospital Israelita Albert Einstein, São Paulo, Brazil; Department of Internal Medicine, University of Iowa Carver College of Medicine, Iowa City, IA USA

**Keywords:** Antimicrobial therapy, Empirical therapy, Infectious diseases

## Abstract

**Background:**

There is universal awareness of the difficulties faced by doctors when prescribing antimicrobials.

**Methods:**

Over a six-month period patients hospitalized in the ICU and under treatment with antibiotics and/or antifungals were eligible to participate in the study. The data were assessed by two infectious diseases specialists. Once completed, all case forms were sent independently to both evaluators (TZSC and ARM) by e-mail. Based on the data received, the evaluator completed a form automatically generated on the e-mail and returned it to the original mailbox for further analysis. We assessed the level of agreement between infectious disease specialists and the physicians directly responsible for the decision to begin antimicrobial therapy, as well as to assess the appropriateness of the regimen prescribed.

**Results:**

Among the antimicrobial regimens prescribed to the 177 patients, 36 % were considered inappropriate by specialist #1 and 38 % were considered inappropriate by specialist #2. We found 78 % agreement by at least one of the infectious disease specialists with the prescribed antimicrobial regimen, and in 49 % of cases both specialists agreed with the prescribed regimen. Both disagreed with the prescribed regimen in 22 % of the cases and they disagreed between themselves in 29 % of the cases.

**Conclusion:**

This study highlights the difficulties in prescribing effective empirical antimicrobial therapy - they are of such magnitude that even two specialists in infectious diseases, well acquainted with our hospital’s resistance patterns and our patients’ profiles have considerable disagreement.

## Background

Infections are a major risk factor for unfavorable clinical outcomes, particularly in the critical care setting [1–5], where the majority of patients receive antimicrobial therapy [1, 2]. Appropriate antimicrobial therapy may help reduce mortality in critically ill patients, and each hour of delay in the administration of the appropriate antimicrobial agent is associated with a 7.6 % increase in mortality [6–9]. Therefore, empiric broad-spectrum antimicrobial coverage is recommended in the more severe cases, particularly when multidrug-resistant microorganisms are suspected [1, 6, 7, 10].

While antimicrobial drugs have changed the prognosis of such patients, their excessive and indiscriminate use has led to the development of resistant microorganisms, infection with *Clostridium difficile,* and other adverse events [1, 3, 6, 11–18].

Several factors allow for widespread use of antimicrobial agents even when inappropriate. A major factor is unrestricted availability to antimicrobials [12, 13], combined with social, cultural and behavioral factors, and certain institutional policies [13]. Also, broad-spectrum empirical therapy is common, even when clinical signs of infection are absent [19].

In order to limit antimicrobial-induced selective pressure, especially that resulting from the use of broad-spectrum antibiotics [4, 6, 10, 17], a de-escalation approach (a change in antibiotic therapy to a narrower spectrum regimen compared to the initial antibiotic prescription) has been encouraged; however, it is not a commonly performed medical practice [1, 6, 7, 10, 18].

The objective of this study was to assess the level of agreement between infectious disease specialists and the physicians directly responsible for the decision to begin antimicrobial therapy, as well as to assess the appropriateness of the regimen prescribed.

## Methods

The study was conducted in the medical-surgical Intensive Care Unit (ICU) of Hospital Israelita Albert Einstein, in São Paulo, SP, Brazil, from June 2010 through December 2011. All 40 ICU beds were included, most of them occupied by private patients; a small percentage of these beds are reserved for patients from the public healthcare network, usually those receiving transplants. The ICU operates with an open staffing model, and the prescription of antimicrobials, as well as other medical decisions, generally reflects the interactions between the specialists involved in each case and the ICU staff physicians, rather than being the sole responsibility of the primary team. However, ultimate decisions are made by the primary physicians responsible for the patient (not the ICU physician that also participates in the care of the patient).

This was a prospective, observational study approved by the IRB of Hospital Israelita Albert Einstein. The requirements for informed consent were waived by our IRB in accordance of the Code of Federal Regulation and of the Privacy Rule. Epidemiologic and laboratory data were captured on a case report form (CRF); the collected information was considered sufficient by the study team to assess the prescription of antimicrobials in the hospital’s ICU.

Patients hospitalized in the ICU and under treatment with antibiotics and/or antifungals were eligible to participate in the study, provided they met the following criteria: CRFs were completed within 72 h of the antimicrobial introduction; age > 18 years; antimicrobial prescription occurred after patient’s admission to the ICU. Patients admitted to any other hospital ward were eligible to participate if the antimicrobial had been prescribed immediately before the patient’s transfer to the ICU. The exclusion criteria were: patients receiving antimicrobial prophylaxis post-operatively and/or due to immunosuppression; >72 h elapsed from the start of antimicrobial therapy; antimicrobials were prescribed outside the ICU, except in those cases described above; and the early postoperative period following organ transplantation.

Data on “recent hospital admission” (within past 3 months) and “prior use of antibiotic therapy” (past 15 days) were obtained from patients, family members, caregivers and/or verified in the patient’s medical records. After the time of admission, all information was obtained exclusively from the patient’s medical records.

Two different procedures for data collection were adopted. For newly admitted patients, if the antimicrobial was prescribed at admission, data were obtained exclusively from the hospital admission forms (physician and nurse), and subsequent lab data were disregarded; if the antimicrobial was introduced after laboratory data were available, the admission forms and the lab report were collected. For patients already hospitalized and who had their antimicrobial regimen newly prescribed and/or changed according to the above mentioned criteria, data were collected from the 24-h period prior to the introduction/change in the regimen; for the purposes of analysis, the most extreme laboratory data were considered.

Organ dysfunctions were diagnosed based on the following measurements: cardiovascular dysfunction: systolic BP <90 mmHg or mean BP <65 mm Hg or use of vasopressors (noradrenaline, dopamine, epinephrine, vasopressin); hematologic disorders: platelets <100,000; liver dysfunction: total bilirubin >2.0 mg/dL; neurological disturbance: drowsiness, confusion, agitation or coma; renal disorders: creatinine >2.0 mg/dL or urine output <0.5 mL/kg/h over the previous 6 h or indication for hemodialysis (except chronic maintenance dialysis); respiratory dysfunction: O_2_ saturation <90 % with or without O_2_ supplementation or pO_2_/FiO_2_ < 200. Tissue hypoperfusion was quantitatively measured by serum levels of arterial lactate [7, 20].

Previous antibiotic treatment was defined as an antibiotic prescribed for at least 48 h during the fifteen-day period prior to the onset of the current antimicrobial therapy [20].

Patients undergoing long-term steroid therapy, chemotherapy and/or radiation therapy, immunosuppressant therapy due to organ transplantation, and those with neoplasms were considered to be immune compromised.

Patients were considered institutionalized if they had been transferred from nursing homes or equivalent facilities.

### Specialists’ opinion

The data were assessed by two infectious diseases specialists with 10- and 17-years experience in the field, both holding master degrees and one of them also a PhD. Both have been members of the hospital’s Nosocomial Infections Control Commission and have a solid reputation in their field; additionally they are routinely involved in the care of critically ill patients admitted to the ICU.

Once completed, all case forms were sent independently to both evaluators (TZSC and ARM) by e-mail. Based on the data received, the evaluator completed a form automatically generated on the e-mail and returned it to the original mailbox for further analysis. On this form, the specialist was required to say if he agreed or disagreed with the prescribed antimicrobial regimen. If he said “no”, he was instructed to justify his disagreement; for example, the regimen was broader than necessary, at least one class of antimicrobial was unnecessary, the regimen was narrower than necessary, or there was no indication for an antimicrobial agent.

### Statistical analysis

This is a descriptive study; the only statistical test applied was the *chi*-square test, at the significance level of *p* = 0.05. Statistical analyses were done using the Statistical Package for the Social Sciences software (SPSS, Chicago, IL, U.S.A.). For all other tabulations we used Microsoft Excel.

## Results

In this study, 177 patients were assessed (100 [56.5 %] males and 77 [43.5 %] females). Sixty one percent of the patients were older than 60 years (65.09 ± 20.35). Thirty-five percent were immune compromised, mostly due to transplantation and/or steroid therapy. Most patients (62.1 %) had a prior history of antimicrobial therapy at the time of data collection and approximately one third had a previous recent hospital admission. There was evidence of at least one organ dysfunction in 78 % of the patients, confirming the severity of these cases. Table 1 shows the data obtained.

**Table 1:** Patient characteristics

		n (177)	(%)
Gender			
	Male	100	56.5
	Female	77	43.5
Age range			
	<20	7	4.0
	20 – 30	5	2.8
	30 – 40	9	5.1
	40 – 50	16	9.0
	50 – 60	31	17.5
	60 – 70	33	18.6
	70 – 80	24	13.6
	80 – 90	35	19.8
	>90	17	9.6
Comorbidities			
	None	14	7.9
	Systemic arterial hypertension	43	24.3
	Diabetes mellitus	37	20.9
	Cerebrovascular accident	22	12.4
	Chronic obstructive pulmonary disease	20	11.3
	Chronic renal insufficiency	19	10.7
	Liver cirrhosis	19	10.7
	Hypothyroidism	16	9.0
	Dementia (Alzheimer/Vascular)	14	7.9
Immunocompromised			
	Yes	62	35.0
	No	115	65.0
Institutionalized			
	Yes	5	2.8
	No	172	97.2
Hospital stay less than 30 days			
	Yes	54	30.5
	No	123	69.5
Previous antibiotic treatment			
	Yes	110	62.1
	No	67	37.9
Clinical signs of SIRS			
	Hypo or hyperthermia	40	22.6
	Tachycardia	102	57.6
	Tachypnea	137	77.4
Devices			
	Only indwelling urinary catheter	15	8.5
	Only central venous catheter	26	14.7
	Central venous catheter and indwelling urinary catheter	53	29.9
Surgery within past 7 days			
	Abdominal	13	7.3
	Neurosurgery	14	7.9
	Orthopedic	2	1.1
	Thoracic	3	1.7
	Thoracic-abdominal	1	0.6
	Liver transplant	5	2.8
Organ dysfunction			
	None	39	22
	One	65	36.7
	Two	35	19.8
	Three	19	10.7
	Four	12	6.8
	≥5	7	4.0
Change in therapy regimen upon culture result			
	Yes	29	16.4
	Respiratory culture	12	6.8
	Urine culture	8	4.5
	Blood culture	5	2.8
	Other	4	2.2

SIRS – Systemic Inflammatory Response Syndrome

We found 78 % agreement by at least one of the infectious disease specialists with the prescribed antimicrobial regimen, and in 49 % of cases both specialists agreed with the prescribed regimen. Both disagreed with the prescribed regimen in 22 % of the cases and they disagreed between themselves in 29 % of the cases (Table 2).

**Table 2:** Analysis of agreement in antimicrobials prescription

	n (354)	%
Mutual agreement (both specialists agreed) with prescribing physician	172	48.6
Agreement by at least one specialist with prescribing physician	276	78.0
Mutual disagreement (both specialists disagreed)	78	22.0
Disagreement between specialists	104	29.4

Among the antimicrobial regimens prescribed to the 177 patients, 36 % were considered inappropriate by specialist #1 and 38 % were considered inappropriate by specialist #2, as shown in Table 3. Concerning the reasons for disagreement, nearly one third of the cases were deemed to have no indication for antimicrobials (27 % according to specialist #1 vs 33 % according to specialist #2). Concerning the antibiotic spectrum, the overall percentages deemed inappropriate were similar (73 % vs 67 %); however, the reasons for disagreement were different. Specialist #1 believed that the spectrum of activity was broader than necessary in 19 % of the cases, and in 30 % too narrow. Specialist #2 believed that the spectrum was too broad in 25 % of cases and too narrow in 13 %. Antimicrobial therapy was considered unnecessary in 22 % and 25 % of cases for specialists 1 and 2, respectively. In a small percentage of cases a broader than necessary spectrum plus an unnecessary class of drugs was mentioned (Table 4).

**Table 3:** Inappropriate antimicrobial regimen, per specialist

		Specialist #1		Specialist #2	
	n total	n	%	n	%
Inappropriate	177	63	35.6	67	37.9

**Table 4:** Reasons for assessing antimicrobial therapy as inappropriate

		Specialist #1		Specialist #2	
		n (63)	%	n (67)	%
Antimicrobial activity		46	73.0	45	67.2
	Spectrum too broad	12	19.0	17	25.4
	Spectrum too necessary	19	30.2	9	13.4
	Unnecessary antimicrobial class	14	22.2	17	25.4
	Broader spectrum and unnecessary antimicrobial	1	1.6	2	3.0
No indication for antimicrobial		17	27.0	22	32.8

In 52 cases, the specialists disagreed between themselves (Table 5); specialist #1 found that the regimen was inadequate in 24 cases and specialist #2 found it inadequate in 28 cases. When we assessed only this sample, we noticed that the major factor explaining the diverging opinions was the spectrum - either more broad or more narrow than necessary; specialist #1 judged 12 cases as too narrow (50 %) vs 1 case too broad (4 %) while specialist #2 judged 2 cases as too narrow (7 %) vs 8 as too broad (29 %), suggesting a more conservative profile of the latter.

**Table 5:** Reasons for discordance between specialists when antimicrobial therapy was deemed inappropriate

	Specialist #1		Specialist #2	
	n (24)	%	n (28)	%
Spectrum too narrow	12	50.0	2	7.1
Spectrum too broad	1	4.2	8	28.6
Unnecessary class	5	20.8	7	25.0
No indication for antimicrobial	6	25.0	11	39.3

There was less difference of opinion among the specialists with regards to no indication for antimicrobials (6 cases (25 %) according to specialist #1 vs. 11 (39 %) according to specialist #2). The rate of disagreement with regards to unnecessary class of antimicrobials was small (5 cases (21 %) according to specialist #1 vs. 7 (25 %) according to specialist #2, Table 5).

When we compared the patients’ characteristics between the diverging and the converging samples, only the presence of an indwelling urinary catheter showed a statistically significant difference (*p* = 0.043). There was no difference between specialists on the basis of patient factors (immunocompromised status, *p* = 0.93; previous use of antibiotic, *p* = 0.66; recent surgery, *p* = 0.13; and organ dysfunction, *p* = 0.08).

In the 52 cases where the specialists disagreed between themselves, diagnoses were limited to four groups (Table 6): empiric use (empiric introduction and/or change of the antimicrobial regimen without initial identification of the infection site), sepsis, acute respiratory failure (ARF), and antimicrobial regimen changed after a positive culture was reported. The antimicrobial spectrum was considered broader than necessary or the drugs were considered unnecessary in many cases from the first two groups by specialist #2 and in most cases from the fourth group according to specialist #1. In the ARF group, disagreement was based mainly on a spectrum deemed broader than necessary or an unnecessary antimicrobial class. The most common previously used antimicrobials were glycopeptides (36.4 %) and 3^rd^ generation cephalosporins (31.8 %) (Table 7).

**Table 6:** Main diagnosis in the specialists’ divergent sample (*n* = 52 patients)

Diagnosis	Disagreement between specialists (n)	Reason
Empirical	14	Specialist #1 assessed that in 4 cases the spectrum was too narrow; specialist #2 considered that in 8 cases there was no indication for antimicrobials and in 2 cases the antimicrobial class was unnecessary
Sepsis	9	Specialist #1 assessed that in 4 cases the spectrum was too narrow; specialist #2 assessed in 1 case the spectrum was too narrow, in 3 cases it was too broad and in 1 case the antimicrobial class was unnecessary
ARF	6	Specialist #1 assessed that in 1 case there was no indication for antimicrobial and in 1 case the antimicrobial class was unnecessary. Specialist #2 assessed that in 2 cases the spectrum was too broad and in 2 cases the antimicrobial class was unnecessary
Positive culture	7	Specialist #1 assesssed that in 2 cases the spectrum was too narrow, in 1 case the antimicrobial class was unnecessary, in 1 case the spectrum was too broad, and in 3 cases there was no indication for antimicrobials

*ARF* acute renal failure

**Table 7:** Previously used antimicrobials

Previously used antimicrobials		N (110)	(%)
	Carbapenems	22	20.0
	2nd generation cephalosporin	5	4.5
	3rd generation cephalosporin	35	31.8
	4th generation cephalosporin	15	13.6
	Glycopeptides	40	36.4
	Beta-lactamase inhibitor	20	18.2
	Macrolides	14	12.7
	Quinolones	25	22.7
	Monotherapy	39	35.5
	Combined therapy	71	64.5

## Discussion

We found considerable differences between the specialists’ opinions about the prescribed antimicrobial therapy, with less than half of the prescriptions being judged appropriate by both (Table 2). In general it appears that specialist #1 has a more aggressive approach to the use of antimicrobials, while specialist #2 was more conservative. We could not distinguish a homogeneous pattern justifying such different approaches; however, when we analyzed some diagnostic subsets within the sample with diverging opinions, we noticed that specialist #1 was more aggressive with respect to those subgroups where the patients had sepsis or where the antimicrobial therapy had been started empirically, and more conservative when treating the subgroup in which the introduction or change in antimicrobial therapy had been motivated by a positive culture, where he considered the regimen to be broader than necessary or even unnecessary. It is difficult to understand the reasons for this divergence. In the analysis of the data, we found no other characteristics that might clarify this issue or reveal the nature of the differences in prescription patterns between the two specialists. The act of prescribing depends on a series of variables: medical knowledge, doctors’ and patients’ cultural beliefs, socio-economic factors, a desire to make independent decisions, expectations about the outcome, ability to break the inertia of routine practice and implement recommendations, medical hierarchy and respect for peers [1, 15]. Factors like no familiarity with or no awareness of the guidelines as well as insufficient knowledge about infectious diseases, potential causative agents and local susceptibility may contribute negatively at the moment of prescribing these drugs [1, 13]. Furthermore, there is a prevailing feeling of safety and comfort with the use of broad-spectrum empirical therapy, irrespective of the guidelines [13]; in addition, certain doctors overestimate their patients’ expectations about antimicrobials and tend to prescribe these drugs as a means of strengthening a good doctor-patient relationship [21], or to maintain collegial relationships between consultant physicians and the primary service physicians.

In 29 % of the cases the specialists disagreed with each other, which may be explained by individual professional characteristics [13, 22], differing choices of empirical therapy, experience and years of practice, and difficulties of using more sensitive microbiological methods for diagnosis. The development of techniques and resources that enable a faster and more accurate identification of microorganisms and their susceptibility profile is warranted [14, 17]. The prior administration of an antimicrobial agent is the most important risk factor for drug-resistant nosocomial infections since it predisposes the patient to colonization by bacteria that are usually resistant to that agent [3, 9, 23]. In our study, 62.1 % of the patients had prior treatment with some form of antimicrobial therapy: 20 % received carbapenems, 36 % glycopeptides and 64.5 % received combination therapy (Table 7). Some studies indicate that over one-third of hospitalized patients receive at least one antimicrobial during hospitalization [15], and among critically ill patients this rate exceeds 70 % [1, 2].

Some strategies seem to be effective in the fight against the increase of resistant microorganisms and the indiscriminate use of antimicrobials. These include restriction and pre-approval forms that limit the availability of antimicrobials [6, 12, 13], audits plus interventions [6, 16], an established prevention policy with more rational use of these agents, i.e., fewer antimicrobials and shorter treatment duration [4, 6, 13, 16], microbiological surveillance [11], review of prescriptions and emphasis on appropriate use by the clinical pharmacist [13, 16], and implementation of an automated system for prescription of antimicrobials [4, 22, 23]. Behavioral approaches to optimize the prescription of antimicrobials do not seem to have the expected result [15, 18], i.e. restrictive interventions are shown to be more effective than those based solely on education and orientation [16, 17].

This study has some limitations, namely the fact that it was a single-center study and data were randomly collected, i.e., not all patients who met the criteria were included. Also, due to the method used for inclusion, some patients with an indication for antimicrobial therapy and who remained untreated were not included in the sample used to evaluate the agreement with the prescription. Furthermore, the study design is not the best recommended for this type of assessment, since the specialists were asked to judge the prescription as appropriate or inappropriate, and the specialist may have been biased by the decision of the prescribing doctor. An alternative approach would have been to ask the specialist to examine the patients’ records and prescribe the antimicrobial regimen without reviewing the drugs that were actually prescribed. Another difficulty with the study when comparing the assessment by the two specialists is the lack of a gold standard. This would have been more easily accomplished should 100 % of our cultures yielded a microorganisms and an antibiogram on which to make a decision (adequate or not adequate antimicrobial therapy). However, there is a low rate of positivity of blood cultures in this population [24], which leads to diversity in antimicrobial practices.

While these data may not be entirely generalizable, they highlight the difficulties in prescribing effective empirical antimicrobial therapy - they are of such magnitude that even two specialists in infectious diseases, well acquainted with our hospital’s resistance patterns and our patients’ profiles have considerable disagreement.

To minimize such differences we need faster and more accurate diagnostic tests as well as a better understanding by frontline providers of clinical guidelines and local susceptibility patterns. Only then can we reach a higher rate of agreement, and implement rational and appropriate use of antimicrobials. Otherwise, in the future we may have further selection of resistant microorganisms against which we scarcely have effective therapeutic resources.
